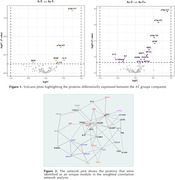# CSF and Plasma Proteomics in the AT groups. What is beyond amyloid and p‐tau?

**DOI:** 10.1002/alz.088515

**Published:** 2025-01-09

**Authors:** Andrea L. Benedet, Nicholas J. Ashton, Ilaria Pola, Marco Antônio de Bastiani, Wagner Scheeren Brum, Nesrine Rahmouni, Stijn Servaes, Jenna Stevenson, Joseph Therriault, Tharick A. Pascoal, Kaj Blennow, Eduardo R. Zimmer, Pedro Rosa‐Neto, Henrik Zetterberg

**Affiliations:** ^1^ University of Gothenburg, Gothenburg Sweden; ^2^ Institute of Neuroscience and Physiology, Sahlgrenska Academy at the University of Gothenburg, Gothenburg Sweden; ^3^ Department of Psychiatry and Neurochemistry, Institute of Neuroscience and Physiology, The Sahlgrenska Academy, University of Gothenburg, Mölndal Sweden; ^4^ Universidade Federal do Rio Grande do Sul, Porto Alegre, Rio Grande do Sul Brazil; ^5^ McGill University, Montreal, QC Canada; ^6^ Translational Neuroimaging Laboratory, The McGill University Research Centre for Studies in Aging, Montréal, QC Canada; ^7^ University of Pittsburgh, Pittsburgh, PA USA; ^8^ Institute of Neuroscience and Physiology, The Sahlgrenska Academy at the University of Gothenburg, Mölndal Sweden; ^9^ Universidade Federal do Rio Grande do Sul, Porto Alegre Brazil; ^10^ Department of Psychiatry and Neurochemistry, Institute of Neuroscience and Physiology, The Sahlgrenska Academy, University of Gothenburg, Mölndal, Gothenburg Sweden

## Abstract

**Background:**

The research on Alzheimer’s disease (AD) has substantially advanced in relation to plasma biomarkers, such as pTau217, for the detection of amyloid (Aβ) pathology which identify, with high accuracy, individuals in the AD biological continuum. However, as these biomarkers become abnormal very early in the disease, biomarkers identifying more advanced disease stages and proxying pathophysiological processes beyond amyloidosis are still needed. Therefore, we have conducted a proteomic study, on plasma and CSF, aiming at identifying proteins reflecting pathological changes in AD.

**Methods:**

Participants from the TRIAD cohort, including CU, MCI or dementia, and with available Aβ and tau PET data, had their plasma samples analyzed with NULISAseq CNS panel (Alamar Biosciences). Participants were grouped according to their Aβ ([^18^F]‐AZD4694>1.55) and tau ([^18^F]‐MK6240>1.19) statuses in AT groups (A‐T‐ (n=242), A+T‐ (n=27) and A+T+ (n=113); discordant participants were not evaluated (n=28)). LIMMA models evaluated the differential protein expression between groups adjusting for age and sex. Additional WGCNA was implemented aiming to evaluate protein modules associated with these AT groups.

**Results:**

When comparing protein expression according to the Aβ status on participants without evidence of tau pathology (A‐T‐ vs A+T‐), the LIMMA analysis unveiled proteins previously associated with Aβ pathology such as pTau217, pTau231 and GFAP, with the addition of two interleukins (Figure 1). However, when contrasting tau pathology status amongst Aβ+ individuals, additional proteins were evidenced such as NPTX1 and NPTX2, NEFL, MAPT, IL6 and IL13. The WGCNA analysis identified one significant protein module that negatively correlated with group A‐T‐ and positively correlated with A+T+ group, recapitulating findings from the initial LIMMA analysis (Figure 2).

**Conclusion:**

In this plasma proteomic study, analysis focusing on Aβ+ groups unveiled proteins that have been largely reported as associated with amyloid pathology and proteins related to immune regulation. However, when the addition of tau pathology was accounted for, proteins associated with tau pathology, inflammation, neurodegeneration and synaptic dysfunction were differentially expressed in the peripheral fluid. These findings will soon be complemented with CSF data, and we will be able to confirm the link between central and peripheral proteomics to brain pathology.